# Temporal trends of sulphadoxine-pyrimethamine (SP) drug-resistance molecular markers in *Plasmodium falciparum* parasites from pregnant women in western Kenya

**DOI:** 10.1186/1475-2875-11-134

**Published:** 2012-07-04

**Authors:** Nnaemeka C Iriemenam, Monica Shah, Wangeci Gatei, Anna M van Eijk, John Ayisi, Simon Kariuki, Jodi Vanden Eng, Simon O Owino, Ashima A Lal, Yusuf O Omosun, Kephas Otieno, Meghna Desai, Feiko O ter Kuile, Bernard Nahlen, Julie Moore, Mary J Hamel, Peter Ouma, Laurence Slutsker, Ya Ping Shi

**Affiliations:** 1 Malaria Branch, Division of Parasitic Diseases and Malaria, Center for Global Health, Centers for Disease Control and Prevention, 1600 Clifton Road NE, MS-D67, Atlanta, GA, 30329-4018, USA; 2 Atlanta Research and Education Foundation/VA Medical Center, Decatur, GA, USA; 3 Department of Infectious Diseases, Tropical Medicine and AIDS, Academic Medical Center, University of Amsterdam, Amsterdam, The Netherlands; 4 Center for Global Health Research, Kenya Medical Research Institute, Kisumu, Kenya; 5 Department of Infectious Diseases, Center for Tropical and Emerging Global Diseases, University of Georgia, Athens, GA, USA; 6 Child and Reproductive Health Group, Liverpool School of Tropical Medicine, Pembroke Place, Liverpool, UK

**Keywords:** Malaria in pregnancy, SP resistance, Kenya, *dhfr*, *dhps*

## Abstract

**Background:**

Resistance to sulphadoxine-pyrimethamine (SP) in *Plasmodium falciparum* parasites is associated with mutations in the dihydrofolate reductase (*dhfr*) and dihydropteroate synthase (*dhps*) genes and has spread worldwide. SP remains the recommended drug for intermittent preventive treatment for malaria in pregnancy (IPTp) and information on population prevalence of the SP resistance molecular markers in pregnant women is limited.

**Methods:**

Temporal trends of SP resistance molecular markers were investigated in 489 parasite samples collected from pregnant women at delivery from three different observational studies between 1996 and 2009 in Kenya, where SP was adopted for both IPTp and case treatment policies in 1998. Using real-time polymerase chain reaction, pyrosequencing and direct sequencing, 10 single-nucleotide polymorphisms (SNPs) of SP resistance molecular markers were assayed.

**Results:**

The prevalence of quintuple mutant (*dhfr* N51**I**/C59**R**/S108**N** and *dhps* A437**G**/K540**E** combined genotype) increased from 7 % in the first study (1996–2000) to 88 % in the third study (2008–2009). When further stratified by sample collection year and adoption of IPTp policy, the prevalence of the quintuple mutant increased from 2.4 % in 1998 to 44.4 % three years after IPTp policy adoption, seemingly in parallel with the increase in percentage of SP use in pregnancy. However, in the 1996–2000 study, more mutations in the combined *dhfr*/*dhps* genotype were associated with SP use during pregnancy only in univariable analysis and no associations were detected in the 2002–2008 and 2008–2009 studies. In addition, in the 2008–2009 study, 5.3 % of the parasite samples carried the *dhps* triple mutant (A437**G**/K540**E**/A581**G**). There were no differences in the prevalence of SP mutant genotypes between the parasite samples from HIV + and HIV- women over time and between paired peripheral and placental samples.

**Conclusions:**

There was a significant increase in *dhfr/dhps* quintuple mutant and the emergence of new genotype containing *dhps* 581 in the parasites from pregnant women in western Kenya over 13 years. IPTp adoption and SP use in pregnancy only played a minor role in the increased drug-resistant parasites in the pregnant women over time. Most likely, other major factors, such as the high prevalence of resistant parasites selected by the use of SP for case management in large non-pregnant population, might have contributed to the temporally increased prevalence of SP resistant parasites in pregnant women. Further investigations are needed to determine the linkage between SP drug resistance markers and efficacy of IPTp-SP.

## Background

Malaria during pregnancy persists as a major public health challenge with adverse consequences for the pregnant woman and the developing foetus. About 50 million women living in malaria-endemic countries become pregnant each year [1]. In Africa, an estimated 30 million women are at risk of *Plasmodium falciparum* infection during pregnancy annually [2]. Human immunodeficiency virus (HIV) and malaria have overlapping global distributions [3]. Currently, available interventions to prevent or mitigate the adverse effects of malaria during pregnancy include intermittent preventive treatment in pregnancy (IPTp), insecticide-treated bed-nets (ITNs), and case management. In sub-Saharan Africa, the World Health Organization (WHO) recommends the administration of at least two curative doses of sulphadoxine-pyrimethamine (SP) for IPTp after the first trimester of pregnancy [4]. A recent study indicated that three doses of SP are required to provide adequate protection to pregnant women [5]. More SP doses are also recommended for women with HIV who are not taking daily cotrimoxazole (CTX, sulphamethoxazole-trimethoprim) for the prevention of opportunistic infections [4]. However, for HIV-infected women taking CTX, WHO does not recommend IPTp-SP, as CTX inhibits the same enzymes as SP in the folic acid biosynthetic pathway, presumably acting as an anti-malarial, and the excess of adverse events were observed in the SP and CTX co-medication [4].

In most African countries, SP remains the recommended drug for IPTp due to its safety profile, efficacy, cost effectiveness and easy administration through the existing health structures, such as antenatal clinics (ANC) [6,7]. IPTp-SP has been shown to reduce the prevalence of placental malaria infection, maternal severe anaemia in primigravidae [8-12], preterm delivery [5,13], infant low birth-weight [5,13-15] and the neonatal mortality rate [16]. However, *P. falciparum* resistance to SP has emerged, originating at the Thailand-Cambodia border and then spreading rapidly to other Asian countries and subsequently to Africa [17]. Concurrently, cross-resistance has also developed between trimethoprim and pyrimethamine in *P. falciparum* malaria parasites [18]. Due to the increasing SP and existing high chloroquine resistance, WHO has recommended artemisinin-based combination therapy (ACT) as alternative first-line treatment for uncomplicated malaria in most endemic countries. However, ACT is not recommended for prevention of malaria including in pregnant women due to the absence of adequate safety data [19].

Resistance to SP has been linked to point mutations in the parasite dihydrofolate reductase (*dhfr*) and dihydropteroate synthase (*dhps*) genes [20,21]. Mutations in *dhfr* confer resistance to pyrimethamine, which occurs in a stepwise pattern [22], while mutations in *dhps* confer resistance to sulphadoxine and other sulpha drugs. In sub-Saharan Africa, the *dhfr* triple mutant (Asn-108/Ile-51/Arg-59) and *dhps* double mutant (Gly-437/Glu-540) have been strongly associated with resistance to SP while the Leu-164 mutation, a major contributor to the rapid spread of high level anti-folate resistance, has recently begun to emerge in Africa [23-25]. The *dhps* mutation at codon 581 is linked with a high rate of therapeutic failure and the emergence of the *dhps* triple mutant allele (A437**G**/K540**E**/A581**G**) has been confirmed in Tanzania [26], and the prevalence is increasing [27]. Recently, the *dhps* mutation at positions 581 and 613 were reported in Kenya [28]. Collectively, these data confirm that the quintuple mutant, comprised of the *dhfr* triple mutant (Asn-108/Ile-51/Arg-59) genotype and *dhps* double mutant (Gly-437/Glu-540) genotype, and other additional *dhfr* and *dhps* mutations are good predictors of SP treatment failure in children [25,29-31].

The rapid spread of SP-resistant parasites associated with SP treatment failure in children [31] and paucity of scientific data linking SP resistant parasites and IPTp-SP efficacy in pregnant women [32] highlights the need for an evaluation of the prevalence of SP molecular markers in the context of IPTp-SP. Recently, studies conducted in Tanzania (data and samples from 2002–2005) showed that IPTp-SP was associated with an increased fraction of parasites carrying the resistant allele at *dhps* 581, an increased parasite density and intense placental inflammation [33], and further suggested that IPTp may not improve overall pregnancy outcomes [34]. However, IPTp-SP was effective in preventing placental malaria infection in several subsequent studies conducted in other African countries [5,13,35]. Another recent report showed that IPTp-SP increased the prevalence of *dhfr*/*dhps* quintuple mutant infection in HIV-infected pregnant women, but the women with drug-resistant parasites did not have increased malaria-related adverse clinical outcomes [36]. The results from these divergent studies suggest that although IPTp-SP could select resistant parasites, IPTp-SP remains beneficial in its protection against malaria infection in some African countries [5,13,35].

This study was conducted using the parasites from Kenyan pregnant women to (a) determine the temporal trends of SP resistance molecular markers over 13 years (between 1996 and 2009), (b) assess if there are any notable differences between peripheral and placental parasites in the profile of SP resistance molecular markers, and (c) examine the relationship between SP resistance molecular markers and IPTp adoption and use of SP during pregnancy, further stratified by HIV status. The study was conducted in the malaria endemic area of western Kenya where there is a high prevalence of HIV co-infection and IPTp-SP policy and SP treatment policy were both adopted in 1998.

## Methods

### Study sites and sample sources

This study used the samples collected from three separate hospital-based malaria in pregnancy studies conducted in Nyanza province, western Kenya between 1996 and 2009. Details for these three observational studies have been described elsewhere ([37,38] and Ouma et *al*, manuscript in preparation). Briefly, between 1996 and 2000, a malaria in pregnancy study was conducted in Provincial General Hospital, Kisumu town to assess the effect of placental malaria on perinatal HIV infection. A second malaria in pregnancy immunological study designed to investigate cellular immune responses in pregnant women was carried out at the same hospital from 2002 to the middle of 2004 and then continued at Siaya District Hospital until 2008. A third malaria in pregnancy study was conducted between 2008 and 2009 in both Siaya and Bondo District Hospitals and the aim of this study was to assess the effectiveness of daily IPTp with SP or CTX prophylaxis in preventing placental malaria in pregnant women. Both Siaya and Bondo District Hospitals are located in rural areas around Kisumu town. Compared to Kisumu town, the rural areas have a relatively higher malaria transmission. The first two studies collected the history of self-reported SP use (case management or any dose of IPTp) during pregnancy while the 2008–2009 study obtained information for both IPTp-SP use and CTX use validated by antenatal clinic records. In Kenya, although the policy of IPTp-SP was adopted in 1998 and it remains till now as per Kenya Ministry of Health (MOH) guidelines, uptake was slow, evidenced by the study showing only 7 % of pregnant women received more than one dose of SP in western Kenya 4 years after the adoption of IPTp-SP policy [39]. Daily CTX prophylaxis for the prevention of opportunistic infections in pregnant women was officially recommended as one component of HIV care in 2005 and the distribution of free ITNs to pregnant women became part of antenatal care in early 2008. In addition, although the drug policy in Kenya shifted to SP from chloroquine in 1998 and then to Coartem® in 2004 for the treatment of uncomplicated malaria, as late as 2010, SP was still used to treat uncomplicated malaria in 37 % of households surveyed in Kisumu, compared to 32 % that used ACT [40]. The prevalence of the *dhfr/dhps* quintuple mutant in children from the same study area increased from 29 % in 1996 to 62 % and 85 % in 2001 and 2007, respectively [41].

For all three observational studies described above, samples were collected at delivery. The selection of samples from the three studies for the present investigation was based on the availability of samples in both HIV + and HIV- women rather than randomization of characteristics. In total, 489 peripheral samples from patients who were *P. falciparum* parasite positive at delivery were used for the study: 180 from 1996–2000, 176 from 2002–2008 and 133 from 2008–2009. In addition, among the 133 peripheral samples from the 2008–2009 period, 125 were paired with placental samples from the same woman.

This study was approved by the Institutional Review Board of the Kenya Medical Research Institute (KEMRI) Nairobi, Kenya, the University of Georgia, Athens, USA, and the Centers for Disease Control and Prevention (CDC) Atlanta, Georgia, USA. Written informed consent was obtained from all study participants.

### Laboratory procedures

#### DNA purification

Genomic DNA was purified from dried blood spots collected from the 2008–2009 period using the Chelex method [42] and also from frozen red blood cell pellets for the remaining samples by micro-centrifugation using a commercial DNA purification QIAamp® blood mini-kit (Qiagen Inc., CA, USA).

#### Single nucleotide polymorphisms genotyping

Ten single nucleotide polymorphisms (SNPs) at *dhfr* codons 50, 51, 59, 108 and 164, and *dhps* codons 436, 437, 540, 581 and 613 were typed using real-time polymerase chain reaction (RT-PCR), pyrosequencing and direct sequencing.

#### RT-PCR

RT-PCR (Stratagene Mx3005P, CA, USA) was used to detect SNPs at *dhfr* codons 51, 59, 108, 164 and *dhps* codons 437 and 540 for 2008–2009 samples using a published procedure [43]. Briefly, standards and field samples were run in duplicate in 25 μl reactions containing TaqMan Universal Mastermix (Applied Biosystems, CA, USA), 2 μl of DNA (diluted 1:10), gene-specific forward and reverse primers, and SNP-specific TaqMan MGB probes (Applied Biosystems, CA, USA). Four 10-fold serial dilutions of both wild type and mutant parasite laboratory strain standards, depending on the SNP, and negative control templates were run on every plate as positive and negative controls.

#### PCR for pyrosequencing and pyrosequencing reactions

The remaining SNPs for 2008–2009 samples and all the SNPs for 1996–2000 and 2002–2008 samples were assayed using a published pyrosequencing method [44]. All PCR primers and sequence primers were synthesized at the CDC Biotechnology Core Facility, Atlanta, USA and experiment conditions were followed based on the described procedures [44]. The following laboratory strains were used as controls for detecting wild and mutant strains: 3D7 (wild type) for *dhfr* 50, 51, 59, 108, 164 and *dhps* 436, 581, 613; Dd2 (mutant) for *dhfr* 50, 51, 59 and *dhps* 613; V1/S (mutant) for *dhfr* 108, 164 and *dhps* 613; FCR3 (wild type) for *dhps* 437; D6 (mutant) for *dhps* 436; 3D7 (mutant) for *dhps* 437; PS-FCR (wild type) and PS-PERU (mutant) for *dhps* 540; and K1 (mutant) for *dhps* 581. No DNA templates were included in all reactions as negative controls. The assays were performed on the PSQ 96 plate using PyroMark ID (Biotage AB, Uppsala, Sweden) and the sample genotypes were determined using the PyroMark ID 1.0 SNP software (Biotage AB).

#### Direct sequencing

All moot results and new mutations were confirmed by direct sequencing. *Dhfr* and *dhps* fragments were amplified using nested PCR sequence protocols [45]. Sequencing of the nested purified PCR products were performed using Big Dye Terminator v3.1 cycle sequencing kit on iCycler thermal cycler (Bio-Rad). The reactions were precipitated in 70 % ethanol to clean up dye terminators, rehydrated in 10 ml HiDi formamide and then sequenced on a 3130 ABI Genetic Analyzer (ABI Prism, CA, USA).

#### Genotyping for multiplicity of infection

Size variant alleles of merozoite surface protein 1 (*msp*1) and *msp*2 were determined by nested PCR for the assessment of multiplicity of infection (MOI). The sequences of the oligonucleotide primers used for amplification of the genetic markers and experimental conditions were set based on the published and recommended methods [46,47]. Positive and negative controls were included in each experiment. The nested PCR products were analysed by 3 % agarose gel electrophoresis and visualized by ultraviolet transilluminator after staining with ethidium bromide. The band sizes were estimated using the gel imaging system and the Labworks image acquisition and analysis software v4.6 (UVP BioImaging Systems, CA, USA).

### Definitions

#### Drug-resistant markers

For all 10 SNPs genotyped, each was coded to designate pure (infection with only either wild type or mutant strains) or mixed (infection with both wild and mutant strains where the minor strain was >30 % of major). Mixed infection was considered as mutant. The SP genotypes (*dhfrdhps* and combined *dhfr*/*dhps*) were categorized based on the criteria of Kublin *et al*[29]. *Dhfr* genotype, based on mutations at codons 51, 59, and 108, was classified as wild type, single, double, and triple (pooled triple mixed and pure) while *dhps* genotype (mutations at codons 437 and 540) was classified as wild type, single, and double (pooled double mixed and pure). Combined *dhfr* and *dhps* genotypes (mutations at *dhfr* 51, 59, 108 + *dhps* 437, 540) were defined as wild type, single, double, triple, quadruple, quintuple (pooled quintuple mixed and pure). The quadruple mutants were categorised either as *dhfr* triple + *dhps* single or *dhfr* double + *dhps* double. The quintuple mutants, in either pure or mixed infection, represent infections in which all the five mutations were detected at codons 51, 59, 108, 437 and 540. Furthermore, additional combinations of *dhps* SNPs at codons 436, 437 and 540, at codons 437, 540 and 581, or at codons 436, 437, 540 and 581 were analysed to further evaluate the progression of *dhps* mutations. In this study, the prevalence of SNP mutations and genotypes were estimated [29].

#### Multiplicity of infection

MOI was assessed by using the polymorphic regions of *P. falciparum msp*1 and *msp*2 genes as markers. Since the size variant alleles of *msp*2 was consistently more diverse than *msp*1 in previous studies [46,48] and in the current study, *msp*2 was selected to evaluate MOI. Mean MOI was calculated as the total number of variant alleles (clones) divided by the number of positive samples for the marker gene *msp*2. Mixed infection was defined as individual infection with more than one variant allele (clone).

#### Clinical

As there were differences in the collection of data for SP use in pregnancy for the three different observational studies, the common variable “SP use in pregnancy (yes or no)” was used which represented the history of self-reported SP use by either case management or any dose of IPTp-SP for first two study periods and validated IPTp-SP use for the third study period. Other variables used in analysis included adoption of IPTp policy (yes or no, policy adopted in 1998), Cotrimoxazole use (yes or no, data only collected in third study), maternal age, gravidity (categorized as primigravid, secundigravid, and multigravid), area of residence, haemoglobin level (g/dl) at delivery (<11 g/dl considered anaemic), peripheral and placental parasite density (parasites/μl) determined by standard microscopic examination of blood smears, and confirmed HIV status by standard laboratory serological tests using same algorithm.

### Data and statistical analysis

The objectives of the present study were to determine the prevalence of SP molecular markers in the parasites from pregnant women over time from 1996 to 2009 and to explore potential epidemiological factors affecting the prevalence of SP resistance molecular markers in the pregnant women. Therefore, the analysis strategies included: 1) a descriptive analysis on temporal trend of drug resistant markers, mixed infection and MOI, and comparisons of these parameters between peripheral and placental samples and between HIV statuses of samples used, and 2) an association analysis between mutations in resistance genotypes as the outcome variables and use of SP in pregnancy as the primary exposure variable.

Characteristics of the study population from the three different studies were analysed by Pearson chi-square tests and ANOVA. Parasite density was log transformed prior to statistical analysis. Differences in the prevalence of SNP mutations, *dhfr*, *dhps* and the combined *dhfr*/*dhps* genotypes among the three studies and other subcomponent comparisons were examined by overall chi-square tests or exact chi-square tests for expected cell counts less than five, considering each molecular marker or genotype as independent. The details of the descriptive analysis are described in additional file [see Additional file 1].

To examine the relationship between SP use in pregnancy and *dhfr, dhps,* and combined *dhfr*/*dhps* genotypes, univariable and multivariable logistic regression analyses were performed. As the original study methodologies differed, data from the three studies were not pooled, but instead, analysed separately. In all epidemiological models, SP use in pregnancy was considered the primary exposure of interest and the (1) *dhfr* (N51**I**/C59**R**/S108**N**)*,* (2) *dhps* (A437**G**/K540**E**)*,* or (3) combined *dhfr/dhps* genotypes as three separate outcomes. The final most parsimonious multivariable model in each study was selected after an assessment of regression assumptions, interaction and confounding factors. No interaction terms were included in any final models as all possible two-way interaction terms with the primary exposure of interest were insignificant at the α = 0.05 significance level based on an overall likelihood ratio test. Confounding was assessed based on a +/− 10 % change in odds ratio (OR) estimate, biological plausibility and the results of univariable analysis. Placental parasite density and maternal age were excluded from the final models due to co-linearity with peripheral parasite density and gravidity, respectively. For the 2008–2009 study, CTX use was excluded from the final model due to expected co-linearity with HIV, as CTX is administered to HIV positive women. The final models derived for all three outcomes and for all three studies contained the variables: use of SP in pregnancy, maternal haemoglobin level at delivery, gravidity, logarithmic peripheral parasite density, HIV status, sample collection year, area of residence and number of *msp2* clones. Given the different distributions of outcome genotype profiles for each study period, binary or cumulative logistic regressions were performed for each study separately. The proportional odds assumption was evaluated for cumulative logistic regression models using the score test, where P < 0.05 reflected a violation of the assumption. In addition, to ensure sufficient sample size in each category for analysis, some genotype categories were collapsed in both binary and cumulative logistic regression. The genotype outcome categories and logistic regression models used for each study period are described in detail in additional file [see Additional file 1]. All statistical tests were two-tailed and statistical significance was defined as P < 0.05. Data analysis was performed with IBM SPSS Statistics version 17.0 (SPSS Inc., IL, USA) and SAS version 9.2 (SAS Inc., NC, USA).

## Results

### Characteristics of the study participants

#### Clinical characteristics

Table 1 describes the characteristics of the pregnant women whose *P. falciparum* parasite positive samples were used in the current study. Use of SP in pregnancy among the participants differed significantly in the three study periods (P < 0.001), with an overall increase from 9.4 % in 1996–2000 study to 67.7 % in 2008–2009 study. In the 2008–2009 study, 23 % of the falciparum malaria parasite positive participants took CTX. There was a difference in mean age but no difference in the parity distribution among the three different studies and, for all three studies, primigravid women were predominant. Parasite density in the placenta was higher than in the periphery. Although there was no statistical difference in both peripheral and placental parasite density among the study periods, there was higher placental parasite density in the samples from 2008–2009 compared to those from 1996–2000 (P = 0.034). More than half of the pregnant women, irrespective of the study period, were anaemic at delivery. In addition, there was a difference in the proportion of HIV sero-positive samples that were used in this study, with a greater proportion of HIV positive samples used in 1996–2000 compared to in 2008–2009.

**Table 1:** **Characteristics of pregnant women who were*Plasmodium falciparum*positive by study period**

**Characteristics**	**1996-2000**	**2002-2008**	**2008-2009**	**P value**^**a**^
	**n = 180 (%)**	**n = 176 (%)**	**n = 133 (%)**	
Use of SP in pregnancy^b^				
Yes				
No	17 (9.4)	90 (53.6)	90 (67.7)	<0.001
	163 (90.6)	78 (46.4)	43 (32.3)	
Septrin/Cotrimoxazole use				
Yes	-	-	32 (23.1)	-
No	-	-	101 (75.9)	
Mother’s age				
Mean ± SD (years)	20.4 ± 4.1	21.1 ± 4.5	22.3 ± 5.3	0.002
Gravidity				
Primigravid	104 (57.8)	90 (51.1)	72 (54.1)	
Secundigravid	40 (22.2)	41 (23.3)	25 (18.8)	0.50
Multigravid	36 (20.0)	45 (25.6)	36 (27.1)	
Placental malaria (parasites/μl)				
Geometric mean	1688.3*	2212.8	3006.5*	0.10
(95 % CI)	(1220.01 to 2336.2)	(1558.2 to 3142.4)	(1946 to 4644.7)	
Peripheral malaria (parasites/μl)				
Geometric mean	713.1	884.3	683.7	0.56
(95 % CI)	(534.4 to 951.5)	(597.7 to 1308.2)	(463.9 to 1007.7)	
Maternal anaemia at delivery^c^				
<11 g/dl	99 (55.9)	63 (55.3)	90 (67.7)	0.030
>11 g/dl	78 (44.1)	51 (44.7)	43 (32.3)	
HIV status				
HIV-	96 (53.3)	120 (68.2)	95 (70.9)	0.002
HIV+	84 (46.7)	56 (31.8)	38 (29.1)	
50				
C (TGT)	180 (100)	176 (100)	132 (100)	N/A
**R** (**C**GT)	0 (0)	0 (0)	0 (0)	
51				
N (AAT)	38 (21.1)	3 (1.7)	3 (2.3)	<0.001
**I** (A**T**T)	142 (78.9)	173 (98.3)	130 (97.7)	
59				
C (TGT)	87 (48.3)	26 (14.8)	13 (9.8)	<0.001
**R** (**C**GT)	93 (51.7)	150 (85.2)	120 (90.2)	
108				
S (AGC)	4 (2.2)	0 (0)	0 (0)	0.031
**N** (A**A**C)	176 (97.8)	176 (100)	133 (100)	
164				
I (ATA)	180 (100)	175 (99.4)	131 (99.2)	0.54
**L** (**T**TA)	0 (0)	1 (0.6)	1 (0.8)	
436^**&**^				
S (TCT)	159 (88.3)	171 (97.2)	124 (93.9)	0.004
**A**/**F**/**H** (**G**CT/**TT**T/**CA**T)	21 (11.7)	5 (2.8)	8 (6.1)	
437				
A (GCT)	103 (57.2)	4 (2.3)	0 (0)	<0.001
**G** (G**G**T)	77 (42.8)	172 (97.7)	133 (100)	
540				
K (AAA)	124 (68.9)	6 (3.4)	1 (0.8)	<0.001
**E** (**G**AA)	56 (31.1)	170 (96.6)	132 (99.2)	
581				
A (GCG)	180 (100)	176 (100)	124 (94.7)	<0.001
**G** (G**G**G)	0 (0)	0 (0)	7 (5.3)	
613				
A (GCC)	178 (100)	176 (100)	133 (100)	N/A
**T**/**S** (**A**CC/**T**CC)	0 (0)	0 (0)	0 (0)	

**Note:** Data are proportion (%) of *Plasmodium falciparum* smear-positive samples unless otherwise indicated. Mutated amino acids and genetic codes are depicted in bold font.

n = number of samples. CI = confidence interval. SD = Standard deviation. N/A = not applicable.

^a^ P values based on Pearson chi-square test or exact chi-square for categorical variables and ANOVA for comparison of the mean of continuous variables.

^b^ For 1996–2000 and 2002–2008 (n = 168) studies, only history of SP use (case management or any dose of IPTp-SP) in pregnancy was documented while IPTp use was recorded for 2008–2009.

^c^ For 1996–2000 (n = 177), 2002–2008 (n = 114).

*Placental parasite density comparison between 1996–2000 and 2008–2009 (P = 0.034).

^**&**^S436H, the new mutation at codon 436, was detected at 2.3 % in 2002–2008 and 3.8 % in 2008–2009.

#### Prevalence of SNP mutations

Mutated amino acids and genetic codes are shown in bold font in Table 1. No mutations were detected at *dhfr* 50 and *dhps* 613 in any study period. However, the mutation at *dhfr* 164 was detected only in one sample each in 2002–2008 (0.6 %) and in 2008–2009 (0.8 %). In addition, the mutation at *dhps* position 581 was not detected prior to 2008, but was found in 5.3 % of the 2008–2009 samples. The prevalence of *dhps* 436 mutations including S436**A**, S436**F**, and S436**H** was 11.7 %, 2.8 % and 6.1 % for the three studies, respectively, among which the new 436 mutation, S436**H**, was detected in 2.3 % of 2002–2008 and 3.8 % of 2008–2009 samples. Overall, the prevalence of SNP mutations in *dhfr* 51, 59, 108 and *dhps* 437, 540 differed significantly among the three studies (P < 0.031), noticeably increasing from 78.9 % to 97.7 % (N51**I**), 51.7 % to 90.2 % (C59**R**), 97.8 % to 100 % (S108**N**), 42.8 % to 100 % (A437**G**) and 31.1 % to 99.2 % (K540**E**), respectively from 1996–2000 to 2008–2009 (Table 1).

### Comparison of the prevalence of SP resistance markers, mean MOI and the number of clones between peripheral and placental samples

**Figure 1:**
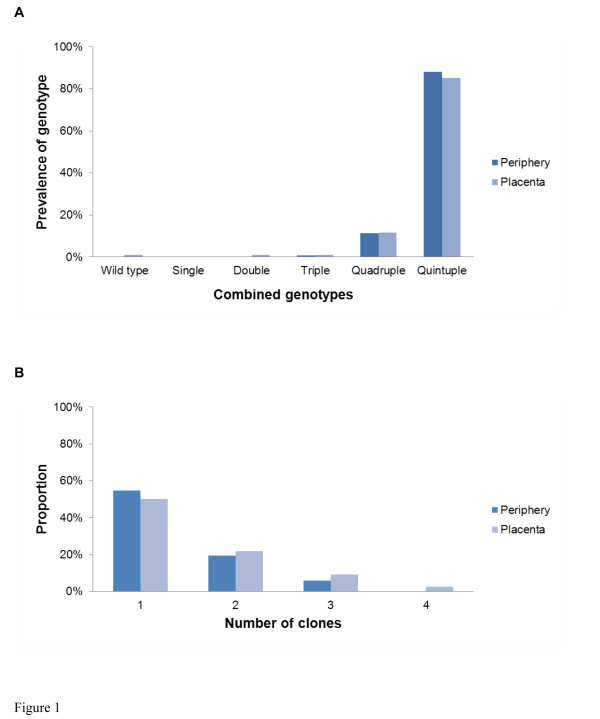
**Comparison of molecular profiles between paired peripheral and placental samples in 2008–2009 study period.A,** combined *dhfr*/*dhps* genotypes. **B**, proportion of the number of clones (*msp*2).

In order to assess if there were any notable differences between peripheral and placental parasites, SP-resistant genotypes, mean MOI and the distribution of the number of clones were compared in the peripheral and placental-paired samples from the 2008–2009 study period. There was no significant difference in the prevalence of the combined *dhfr*/*dhps* genotype in the peripheral and placental-paired samples (P = 0.54) (Figure 1A). In the analysis of mean MOI, the difference between periphery (1.11 ± 0.79) and placenta (1.32 ± 0.94) was not significant (P = 0.20). The number of distinguishable *P. falciparum* clones defined by *msp*2 variant alleles ranged between one and four. No differences in the distribution of number of clones were found in the peripheral and placental-paired samples (P = 0.19) (Figure 1B).

### Temporal trends of *dhfr*, *dhps* and the combined *dhfr*/*dhps* genotypes

**Figure 2:**
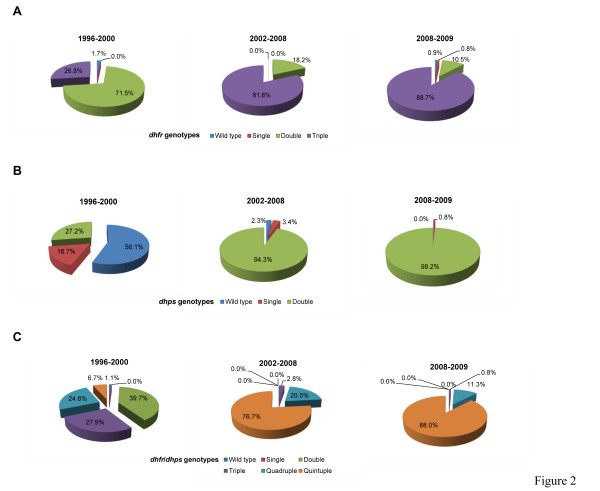
**Temporal trends of SP drug resistant genotypes from 1996 to 2009 by study period.A,***dhfr* genotypes. **B,***dhps* genotypes. **C,** combined *dhfr*/*dhps* genotypes.

Figure 2 shows the temporal trends by the three study periods. The prevalence of the *dhfr* triple mutant (N51**I**/C59**R**/S108**N**) increased considerably from 27 % in 1996–2000 to 89 % in 2008–2009 and was significantly different across studies (P < 0.001), while the *dhps* double mutant (A437**G**/K540**E**) also increased from 27 % to 99 % and also differed significantly among studies (P < 0.001) [Figure 2A, B, respectively]. Figure 2C shows the temporal trends of the combined *dhfr* and *dhps* genotypes, based on the mutations at *dhfr* 51, 59, 108 and *dhps* 437, 540 classified as wild type, single, double, triple, quadruple, and quintuple. The prevalence of the quintuple mutant differed in the three studies (P < 0.001), increasing from 7 % in 1996–2000 to 88 % in 2008–2009. However, in all three studies, there were no statistically significant differences in the prevalence of *dhfr*, *dhps* and the combined *dhfr/dhps* genotypes between the parasites samples from HIV + and HIV- women (see Additional file 2a, b, and c, respectively).

**Figure 3:**
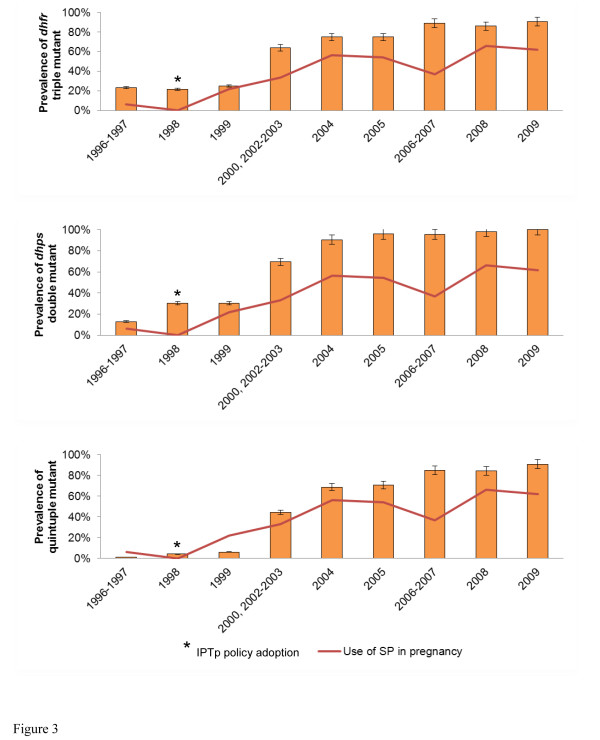
**Prevalence of*dhfr*triple mutant,*dhps*double mutant and the combined*dhfr*/*dhps*quintuple genotype before and after IPTp policy adoption.** Years with fewer than 25 samples were merged with either the previous year or after, depending on the number of samples in that particular year. The bars represent 95 % CI. No samples were collected in 2001 and therefore data for this year was not presented. For 1996–2000 and 2002–2008 periods, only history of SP use (case management or any dose of IPTp-SP) in pregnancy was documented while IPTp use was recorded for 2008–2009.

As the number of years for the three studies varied, particularly the wide range of years (2002–2008) for the second study, the prevalence data was further stratified by years and IPTp policy adoption (Figure 3). The prevalence of *dhfr* triple, *dhps* double and the combined *dhfr*/*dhps* quintuple mutants increased from 22.8 %, 19.5 % and 2.4 % in 1998 to 63.9 % (P < 0.001), 69.4 % (P < 0.001) and 44.4 % (P < 0.001), respectively, about three years after IPTp policy adoption (Figure 3). Subsequently, the prevalence of the drug-resistant molecular markers steadily increased, and by year 2009, the prevalence of the corresponding drug resistance mutant genotypes reached more than 90 % in the study population. In addition, Figure 3 shows that the percentage of participants who used SP during pregnancy after IPTp policy adoption seemingly parallels with the temporal trend for prevalence of drug resistant genotypes.

Table 2 shows the temporal change of three additional *dhps* combinations from 1996 to 2009. The *dhps* triple mutant which consists of the combination of mutations at *dhps* 436, 437 and 540 was not observed in the first study period but increased from 2.3 % in 2002–2008 study to 6 % in 2008–2009 study. In addition, the *dhps* triple mutant comprising of the combination of mutations at *dhps* 437, 540, and 581 was only detected at 5.3 % in 2008–2009 study. Furthermore, the combination of quadruple mutations at *dhps* 436, 437, 540 and 581, not previously detected in Kenya prior to 2008, showed a prevalence of 0.8 % in 2008–2009.

**Table 2:** **Temporal trends of additional*dhps*combinations from 1996 to 2009 by study period**

***dhps*combinations**	**1996-2000** **(%)**	**2002-2008** **(%)**	**2008-2009** **(%)**	**P value**
S436**A**/**F**/**H** + A437**G** + K540**E**				
Wild type	44.4	1.7	0	
Single mutant	25.6	1.7	0.8	<0.001
Double mutant	30.0	94.3	93.2	
Triple mutant	0	2.3	6.0	
A437**G** + K540**E** + A581**G**				
Wild type	56.1	2.3	0	
Single mutant	13.9	1.1	0.8	<0.001
Double mutant	30.0	96.6	93.9	
Triple mutant	0	0	5.3	
S436**A**/**F**/**H** + A437**G** + K540**E** + A581**G**				
Wild type	44.4	1.7	0	
Single mutant	25.6	1.7	0.8	<0.001
Double mutant	30.0	94.3	88.5	
Triple mutant	0	2.3	9.9	
Quadruple	0	0	0.8	

P values derived from exact Pearson chi-square test. Mutated amino acids are depicted in bold font.

### Assessment of association between use of SP in pregnancy and mutations in SP drug resistance genes

Since the increased prevalence in drug resistance molecular markers paralleled the increased use of SP during pregnancy as observed in Figure 3, the association between SP use in pregnancy and its effect on drug resistance molecular markers was further assessed. For the 1996–2000 study only, the unadjusted odds of having the *dhps* double mutant genotype among women who used SP was 3.5 [95 % CI, 1.3 to 9.6] times greater than the odds of women who did not use SP (Table 3). More mutations in the combined *dhfr/dhps* genotype were also associated with SP use during this study period (unadjusted OR, 4.2 [95 % CI, 1.6 to 10.9]). However, after adjusting for all other explanatory variables, neither association remained significant in multivariable analysis (Table 3). No other significant associations between the use of SP in pregnancy and mutations in *dhfr*, *dhps* and the combined *dhfr*/*dhps* genotypes were observed in the 2002–2008 and 2008–2009 study periods.

**Table 3:** Univariable and multivariable analyses of the association between SP use and mutations in SP drug resistance genotypes by study period

**Outcome**	**1996-2000**		**2002-2008**		**2008-2009**	
	Unadjusted	Adjusted^e^	Unadjusted	Adjusted^e^	Unadjusted	Adjusted^e^
	OR (95 % CI)	OR (95 % CI)	OR (95 % CI)	OR (95 % CI)	OR (95 % CI)	OR (95 % CI)
*dhfr* triple mutant^a^						
Use of SP in pregnancy‡	2.7 (1.0 to 7.5)	2.4 (0.8 to 7.1)	1.4 (0.634 to 3.0)	1.4 (0.6 to 3.2)	1.1 (0.3 to 3.3)	1.3 (0.4 to 4.7)
*dhps* double mutant^b^						
Use of SP in pregnancy	3.5 (1.3 to 9.6)*	2.3 (0.7 to 7.0)	1.2 (0.3 to 4.1)	1.3 (0.3 to 5.2)	- ^f^	- ^f^
Combined *dhfr*/*dhps*^c,d^						
Use of SP in pregnancy	4.2 (1.6 to 10.9) *	2.6 (0.9 to 7.4)	1.3 (0.6 to 2.6)	1.4 (0.7 to 2.9)	1.0 (0.3 to 2.9)	1.2 (0.3 to 4.1)

**Note:** OR, odds ratio; CI, confidence interval.

‡For 1996–2000 and 2002–2008 studies, only history of SP use (case management or any dose of IPTp) in pregnancy was documented while IPTp use was recorded for 2008–2009.

^a^*dhfr* triple mutant was compared to the grouped all other *dhfr* genotypes using binary logistic regression.

^b^*dhps* double mutant was compared to the grouped all other *dhps* genotypes using binary logistic regression.

^c^ For 1996–2000, combined *dhfr*/*dhps* mutations were analysed using three categories in cumulative logistic regression: (1) grouping all other genotypes as one group, (2) quadruple mutant, and (3) quintuple mutant.

^d^ For 2002–2008 and 2008–2009, quintuple combined *dhfr*/*dhps* mutant was compared to the grouped all other combined *dhfr*/*dhps* genotypes using binary logistic regression.

^e^ Adjusted ORs are derived from multivariable binary or cumulative logistic regression, controlling for gravidity, log transformed peripheral parasite density, HIV status, sample collection year, and total number of *msp*2 clones.

^f^ Only one sample was single mutant in 2008–2009, while the rest were double mutant, therefore association analysis was not performed.

* Statistically significant, P < 0.05.

### Prevalence of mixed infection and mean MOI over time

**Figure 4:**
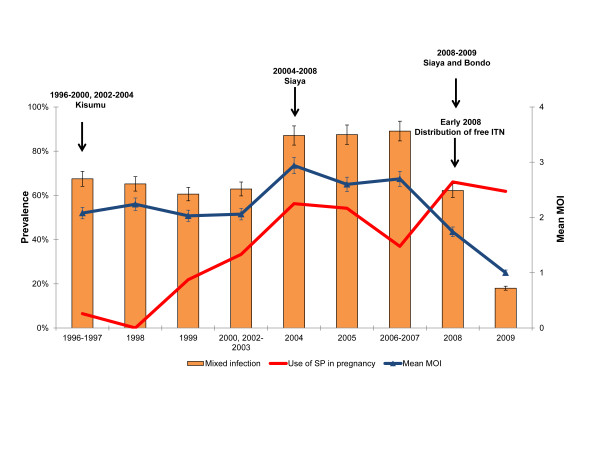
**Prevalence of mixed infection and mean MOI over time.** Years with fewer than 25 samples were merged with either the previous year or after, depending on the number of samples in that particular year. No samples were collected in 2001 and therefore data for this year was not presented. The bars represent 95 % CI while mean MOI error bars represent SD. For 1996–2000 and 2002–2008 periods, only history of SP use (case management or any dose of IPTp-SP) in pregnancy was documented while IPTp use was recorded for 2008–2009.

Previous studies suggest that transmission intensity or transmission reduction by ITNs could be one of factors that influence the prevalence of parasite drug resistance molecular markers [49,50]. In order to estimate the change in transmission intensity during the study period and the possible effect of transmission reduction by ITN intervention on the parasite populations, the prevalence of mixed infection and MOI were measured by using size variant alleles of *msp*2. Figure 4 shows that the prevalence of mixed infections decreased from 67.5 % in 1996–1998 to 18 % in 2009, although the highest prevalence of mixed infections was observed from 2004 to 2007 (87.1 % to 89.1 %, respectively) (P < 0.001). Likewise, the mean MOI also decreased significantly from 2.08 ± 0.96 to 1.00 ± 0.61 between 1996–1998 and 2009 while the highest mean MOI (2.94 ± 1.12 to 2.7 ± 0.99) was detected between 2004 and 2007 period (Figure 4) (P < 0.001).

## Discussion

Anti-malarial drug resistance to SP, a factor contributing to diminishing therapeutic efficacy, is increasing in many malaria endemic countries. All African countries have already replaced SP with artemisinin-based combination therapy as the first line treatment for uncomplicated malaria, according to the WHO guidelines. However, SP remains the recommended drug for IPTp in malaria endemic areas. In this study, the temporal trends of SP resistance molecular markers in the parasites from Kenyan pregnant women and potential epidemiological factors affecting the prevalence of the resistant markers in the pregnant women were evaluated.

The key observations from this study were: 1) a noticeable background level of SP mutations in pregnant women prior to IPTp policy adoption, 2) a significant rise in the prevalence of the drug resistance markers after IPTp adoption seemingly in parallel with an increase in the percentage of SP use in pregnancy, and 3) however an association between more SNP mutations and SP use during pregnancy only in the 1996–2000 study in univariable analysis but not in 2002–2008 and 2008–2009 study periods. These results suggest that the increase in drug-resistant parasites in the pregnant women over time could be influenced by other major factors in addition to the minor role of IPTp adoption and SP use in pregnancy. Firstly, compared to high drug pressure in large general population (children and adults) due to SP use for case management, drug pressure in the small minority of pregnant women by IPTp-SP use is relatively lower. It is most likely that the resistant parasites selected by the use of SP for case management in the large general population could serve as the potential infectious reservoirs for pregnant women, thus high prevalence of drug resistant parasites circulated in the large non-pregnant population could contribute to the observed temporal increase in resistance markers in pregnant women. Indeed, the results from our previous study, conducted in the general population of the same area, showed that the prevalence of *dhfr/dhps* quintuple mutant parasites in children from the same study area increased from 29 % in 1996 to 62 % and 85 % in 2001 and 2007, respectively, after the drug policy change to SP for the treatment of uncomplicated malaria in 1998 [41]. Secondly, IPTp-SP works by intermittently clearing asymptomatic parasitaemia and preventing new infections in pregnant women [51] and the decreasing prevalence and intensity of infection in subsequent pregnancies also indicates the acquired immunity of the pregnant woman in preventing malaria infection as well as clearance of parasites [52]. Recent studies from several African countries reported increased prevalence of mutant genotypes due to IPTp-SP [33,53,54], but the increased resistant parasites did not affect the protective efficacy of IPTp [53,54]. Based on the results from others [53,54] and the molecular data from this study, the authors speculate that the interplay between IPTp-SP and host immunity in pregnant women could also play a role in the selection of drug resistant parasites, consequently shaping the parasite drug resistant profile in pregnant women. However, further studies are required to test this hypothesis. Lastly, the use of CTX as prophylaxis against opportunistic infection in HIV infected pregnant women, particularly the validated CTX use in the third study (2008–2009), might also contribute to the increased prevalence of SP drug resistant genotypes over time, possibly due to cross resistance between trimethoprim and pyrimethamine in *P. falciparum* malaria parasites [18]. Since multiple factors could be involved in the selection of resistant parasites in pregnant women, molecular markers when used alone may not accurately predict the failure of IPTp-SP. The assessment of the linkage between SP drug resistance molecular markers and efficacy of IPTp-SP in pregnant women in different malaria endemic areas becomes extremely important.

This study also detected 5.3 % of the *dhps* triple mutant (A437**G**/K540**E**/A581**G**) (7 samples), the presence of quadruple *dhps* mutant (S436**A**/**F**/**H** + A437**G** + K540**E** + A581**G**) (1 sample), the *dhfr* 164 (I164**L**) mutation (1 sample), and an increase in the new mutation at *dhps* S436**H** (5 samples) in the third study period (2008–2009). These results suggest progressive expansion of new mutations or new genotypes in parasite populations in Kenya. Although the contribution of the new mutation *dhps* S436**H** (unreported prior to this study) to drug resistance is unclear, the mutations at *dhfr* 164 and *dhps* 581 or related genotypes have been associated with a high-level of SP resistance mainly in Southeast Asia and South America [55,56] and, more recently, have been reported on the African continent [26,57-60]. Due to the small sample size in the new drug resistant genotype/SNP group, no further association analyses were conducted. However, higher placental parasite density in 2008–2009 period was observed as compared to the 1996–2000. It is possible that the progressive expansion of the new genotypes could be related to the overgrowth of resistant parasites, i.e. the highly resistant parasites out-compete less fit parasites [61-63]. Recent data from Tanzania suggest that the emergence of the *dhps* triple mutant (A437**G**/K540**E**/A581**G**) associated with the IPTp-SP use in pregnant women led to high-density parasitaemia [33]. Taken together, the emergence of new genotypes containing mutations in *dhps* 436 and *dhps* 581, coupled with the presence of *dhfr* 164 mutation in pregnant women may present a challenge for the future usefulness of IPTp-SP intervention in Kenya.

A recent study conducted in Mozambique reported that IPTp-SP use increased the prevalence of resistance markers in HIV positive women and mutant infections were more prevalent in placental than peripheral samples [36]. However, in this investigation, no statistical differences were found in the prevalence of *dhps* triple, *dhps* double and *dhfr/dhps* combined quintuple mutants between HIV positive and HIV negative women during all the three study periods. In the current study, there was also no significant difference in the prevalence of SP resistance markers and mean MOI between peripheral and placental-paired samples in the third study period when SP resistance was high. The discrepancy of the results between these two studies could be due to the differences in the study design, level of existing SP pressure and resistance, HIV care and other unknown factors. The Mozambique study used samples from a randomized double-blind, placebo-control trial of IPTp-SP conducted before IPTp-SP policy adoption, while the current study employed the samples collected from three different observational studies before and after IPTp-SP adoption for an extended period. However, the observation of no significant differences in mutant infections between placental and peripheral samples in the current study is consistent with the results from the studies conducted in Tanzania and Ghana, the countries with relative high levels of SP resistance [33,64]. This suggests that either peripheral or placental samples can be used for monitoring drug resistant molecular markers in areas with high SP resistance level.

Overall, the prevalence of mixed infection and mean MOI decreased significantly between 1996 and 2009, but the highest parasite diversity was observed between the 2004 and 2007 period. The observed sharp increase in parasite diversity from 2004 could reflect the higher transmission intensity of the study site as the second malaria in pregnancy study was conducted from 2004 in the rural area (Siaya) where malaria transmission is relatively higher compared to Kisumu town. However, the drop in parasite diversity in 2008 followed by a significant decrease in 2009 might be related to the combination of transmission reduction by the implementation of free distribution of ITNs to pregnant women in ANC from early 2008 and SP use in pregnancy. The diversity of *P. falciparum* malaria reflects acquisition of new infections and is associated with transmission intensity [65]. A previous study conducted in Tanzania also showed that parasite diversity measured by *msp2* was reduced in women with recent IPTp-SP use [33]. Nevertheless, the observation, overall decreased parasite diversity in conjunction with increased prevalence of drug resistance molecular markers over time in this study, suggests that intra-host removal of SP drug sensitive parasite clones is present, thus purifying drug resistant parasites in pregnant women.

There were few limitations to the current study. First, the present study utilized data from three different malaria in pregnancy observational studies and these three studies varied in their original study designs. Therefore, there were some differences in patient recruitment and procedures of clinical data collection, including assessment of IPTp use and use of SP for case management, which could have affected the statistical association analysis. Additionally, the lack of baseline samples from women prior to IPTp administration and absence of information for early versus later IPTp-SP or SP use before delivery limited the comparisons to assess the selection of drug resistant parasites in pregnant women whose immunity and pharmacokinetics are altered during pregnancy. Another limitation to the current study was the lack of samples from non-pregnant women as controls from the same study periods due to original design of the three previous observational studies. It will be ideal to conduct a well-controlled longitudinal IPTp drug resistance studies in both pregnant and non-pregnant women of the same population over a period of time. Such studies will help to examine the selection of drug resistant parasites and evaluate the potential factors involved in the development of SP resistance in pregnant women.

## Conclusions

There was a significant increase in *dhfr/dhps* quintuple mutant and the emergence of new genotype containing *dhps* 581 and *dhps* S436**H** in the parasites from pregnant women in western Kenya over 13 years. IPTp adoption and SP use in pregnancy only played a minor role in the increased drug-resistant parasites in the pregnant women over time. Most likely, the high prevalence of resistant parasites selected by the use of SP for case management in large non-pregnant population in the same study area, CTX use, host immunity and transmission intensity might have contributed to the temporally increased prevalence of SP resistant parasites in pregnant women. Further investigations in the region to determine the linkage between SP drug resistance molecular markers and efficacy of IPTp-SP are needed.

## Competing interests

The authors declare that they have no competing interests.

## Author’s contributions

NCI, MS, WG, SK, AAL and YOO carried out genotyping work for the current study. AME, JA, FOK, BN (first period), JM, SOO (second period), MD, MJH, PO, and LS (third period) implemented the observational malaria in pregnancy studies from 1996 to 2009 including enrolment of patients and collection of clinical and epidemiological data and samples. YPS conceived, designed and supervised the current study. NCI, MS and JVE did the statistical analysis. NCI, MS and YPS wrote the paper. All authors contributed to the interpretation of results and critical discussion of the conclusion and approved the final manuscript.

## Supplementary Material

Additional file 1 **Statistical procedure.** The file described the statistical methods used for the descriptive and association analysis. Click here for file

Additional file 2 **Proportion of SP drug resistant genotypes by HIV status and by study period.** A, *dhfr* genotypes. B, *dhps* genotypes. C, combined *dhfr*/*dhps* genotypes. The figures described the prevalence of *dhfr*, *dhps* and the combined *dhfr/dhps* genotypes between HIV + and HIV- women. Click here for file
