# Supplementary material for: Temporal trends of sulphadoxine-pyrimethamine (SP) drug-resistance molecular markers in Plasmodium falciparum parasites from pregnant women in western Kenya
Source: Malar J. 2012 Jul 4;11:134. doi: 10.1186/1475-2875-11-134 (PMC3390272; doi:10.1186/1475-2875-11-134)
Supplement: Additional file 1 — Statistical procedure. The file described the statistical methods used for the descriptive and association analysis. [file 1475-2875-11-134-S1.doc]

**Additional file 1 Statistical procedure**

1. Descriptive analysis

| **Type of analysis** | **Markers** | **Results presented** | **Stratified by** | **Methods used** |
| --- | --- | --- | --- | --- |
|  |  |  |  |  |
| Temporal trends | SP drug resistant markers  *msp*2 | Prevalence of SNP mutations, *dhfr, dhps*, *dhfr/dhps* combined and additional *dhps* genotypes  Percentage of mixed infection and MOI | Study period,  IPTp adoption, and Year | Exact Pearson Chi Square,  ANOVA |
| Comparison of HIV status | *dhfr*, *dhps*, *dhfr/dhps* combined | Prevalence of *dhfr, dhps*, *dhfr/dhps* combined genotypes | Study period | Pearson Chi Square |
| Comparison between paired periphery and placenta | *dhfr*, *dhps*, *dhfr/dhps* combined  *msp*2 | Prevalence of *dhfr, dhps*, *dhfr/dhps* combined genotypes  Percentage of mixed infection and MOI | Only in 2008-2009 | Pearson Chi Square  ANOVA |

1. **Association analysis**

| **Predictor** | **Genotype** | **Genotype outcome categories** | **Study** | **Logistic regression** |
| --- | --- | --- | --- | --- |
| Use of SP in pregnancy | *dhfr* | 1. Wild type, Single, and Double (Reference) 2. Triple | 1996-2000  2002-2008  2008-2009 | Binary |
|  | *dhps* | 1. Wild type and Single (Reference) 2. Double | 1996-2000  2002-2008 | Binary |
|  | Combined *dhfr/dhps* | 1. Wild type, Single, Double, and Triple (Reference) 2. Quadruple 3. Quintuple | 1996-2000 | Cumulative |
|  | Combined *dhfr/dhps* | 1. Wild type, Single, Double, Triple, and Quadruple (Reference) 2. Quintuple | 2002-2008 2008-2009 | Binary |
