# Supplementary figures and images for: Temporal trends of sulphadoxine-pyrimethamine (SP) drug-resistance molecular markers in Plasmodium falciparum parasites from pregnant women in western Kenya
Source: Malar J. 2012 Jul 4;11:134. doi: 10.1186/1475-2875-11-134 (PMC3390272; doi:10.1186/1475-2875-11-134)

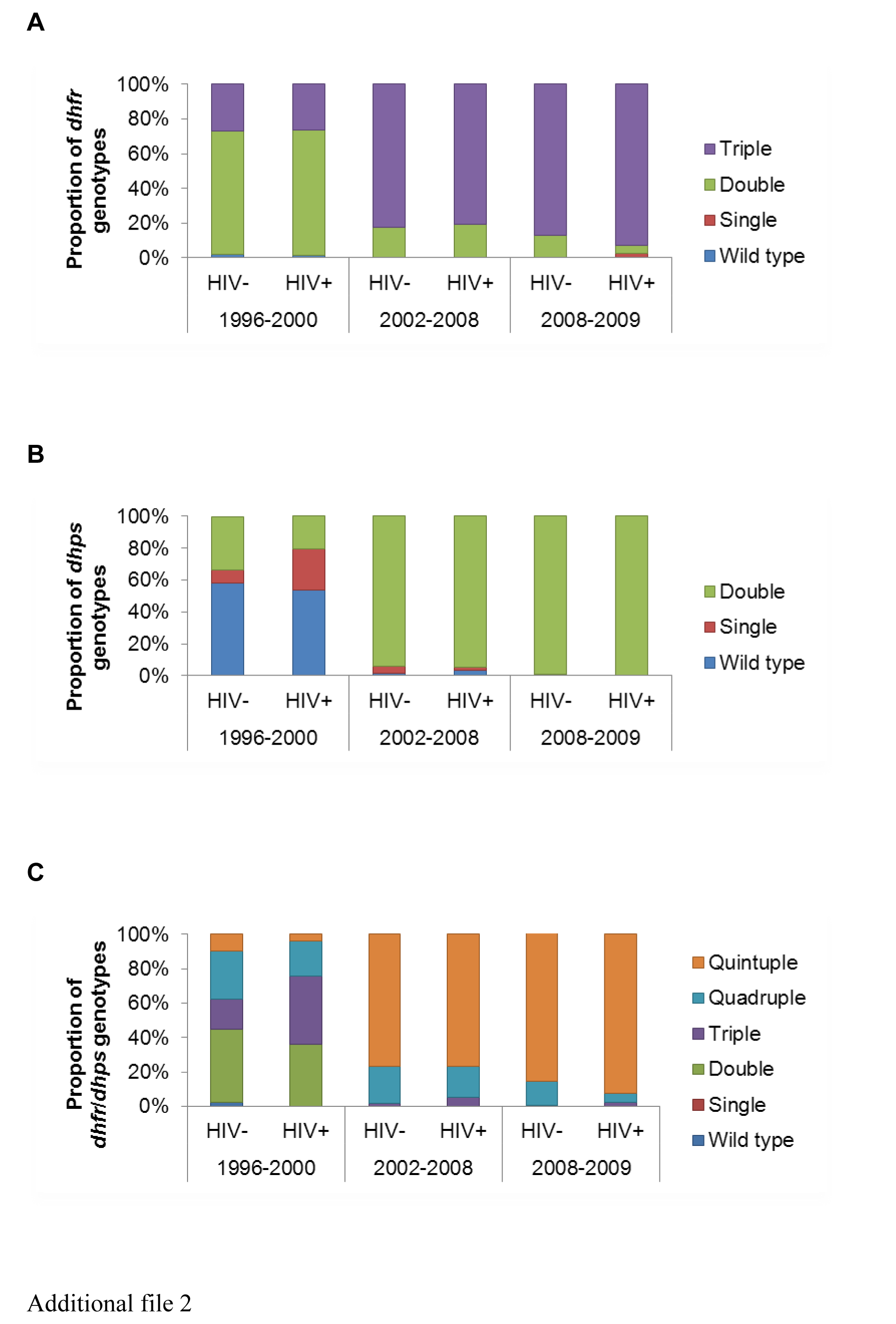

Supplement: Additional file 2 — Proportion of SP drug resistant genotypes by HIV status and by study period. A, dhfr genotypes. B, dhps genotypes. C, combined dhfr/dhps genotypes. The figures described the prevalence of dhfr, dhps and the combined dhfr/dhps genotypes between HIV + and HIV- women. [file 1475-2875-11-134-S2.tiff]
